# Association of Sleep Duration with Arterial Blood Pressure Profile of Gujarati Indian Adolescents

**DOI:** 10.4103/0970-0218.62571

**Published:** 2010-01

**Authors:** Wasim A Shaikh, Minal Patel, SK Singh

**Affiliations:** Department of Physiology, Pramukhswami Medical College, Karamsad-388325, Gujarat, India

**Keywords:** Blood pressure, Gujarati Indian adolescents, sleep duration

## Abstract

**Background and Aim:**

Recently, National Health and Nutritional Examination Survey-1 data analysis found short sleep duration as a risk factor for hypertension in the U.S. population. However, since ethnic differences exist in the aetiopathogenesis of diseases, the current study was undertaken to study the effect of sleep duration on the blood pressure profile of Gujarati Indian adolescents.

**Materials and Methods:**

A cross-sectional study was conducted on 489 Gujarati Indian adolescents of age group 16-19 years studying in school and colleges in the local population. The participants were assessed for their sleep duration, physical activity status, body composition, blood pressure profile and cardiovascular reactivity. The sleep duration was reported by the subjects as the number of hours they slept on most of the nights in a week over the last one year. The observations of the study were then analyzed after grouping them into: 1) Adequate Sleep Duration at Night, ASDN (≥7 hrs) and 2) Inadequate Sleep Duration at Night, ISDN (<7 hrs) groups. Student's unpaired *t*-test was used to study if any significant difference (*P*< 0.05) existed between the groups.

**Results:**

No significant difference was found in Systolic blood pressure, Diastolic blood pressure, Pulse pressure and Mean arterial pressure between the ASDN group and the ISDN group. Physical activity status also did not differ between the two groups. However, adolescents of ISDN group showed a significantly higher level of adiposity and cardiovascular reactivity as compared to adolescents of ASDN group.

**Conclusion:**

Although short sleep duration is associated with a higher level of adiposity and cardiovascular reactivity in Gujarati Indian adolescents, it does not affect the resting blood pressure profile of these adolescents. However, longitudinal studies would be required to observe if the changes in adiposity and cardiovascular reactivity affect these adolescents in later life.

## Introduction

A phenomenal rise has been observed in the prevalence of prehypertension and hypertension among the Indian youth.(1,2) The major factors that have been largely reported for the sustain rise in blood pressure amongst the youth are increasing sedentary lifestyle along with overweight or obesity amongst the Indian youth.(2,3) However, recent studies conducted in various parts of the world indicate short sleep duration as a risk factor for development of prehypertension and hypertension amongst adolescents and adults.(4–6) Sleep deprivation studies have shown that restricted hours of sleep at nights result in significant rise in blood pressure during the day time on the following day. Various mechanisms have been delineated that link short sleep duration with the rise in blood pressure. Elevated sympathetic nervous system activity, waking physical and psychosocial stressors, increased salt retention, obesity and disruption of circadian rhythmicity associated with short sleep duration have been linked to hypertension.(6)

But since there are scant reports available from Indian population, which indicate the influence of sleep on the blood pressure of Indians and the fact that aetipathogenesis differs across ethnicity, it is essential to determine the role of sleep quantity and sleep quality in the pathogenesis of hypertension in the Indian population. The current study was therefore undertaken to assess the effect of sleep duration on blood pressure profile of the Gujarati Indian adolescents. The study was framed keeping the hypothesis that short sleep duration may affect the blood pressure profile of the adolescents by affecting their day time behavior such as physical activity status and/or by affecting their body composition, and/or by affecting their cardiac sympathetic activity at rest and/or cardiovascular reactivity to stress.

## Materials and Methods

A cross-sectional study was conducted from January 2007 to March 2008 after the approval from the human research ethical committee of the institute and obtaining informed consent from the participants or their parent/guardian. Adolescents of age group 16-19 years, both boys and girls, studying in school and colleges in the local population, and who had attained a tanner stage of at least 4 by self-reporting were recruited for the study irrespective of their socioeconomic class.(7) A total of 489 subjects (Boys = 297, Girls = 192) were recruited into the study by multistage sampling.

Sleep duration at night: The participants were asked to self-report the number of hours for which they slept during most of the nights in a week for the last one-year. The subjects reported the sleep duration from the time of going to bed to the time they woke up in the morning. Sleep duration of more than or equal to seven hours per night was considered as Adequate Sleep Duration at Night (ASDN) and sleep duration of less than seven hours was considered as Inadequate Sleep Duration at Night (ISDN).(8,9)

Physical activity status: Participants also reported their physical activity status using the NASA/Johnson Space Center Physical Activity Rating scale.(10)

Body composition: The body composition was assessed in a standardized state of clothing. The body weight (Wt) was recorded bare footed to the nearest 0.5 kg. The height was measured using meter scale without footwear to the nearest 5 cm. BMI was calculated as the weight (kg) divided by the square of height (m^2^). Waist circumference was measured at the midpoint between the lower costal margin and the highest point on the iliac crest to the nearest 0.5 cm at the end of normal expiration.(11,12) Body Fat Percentage (BF%) and Total Body Fat Mass (FM) were assessed by bioelectrical impedance technique using Omron Body Fat Monitor HBF - 302.(13,14) Fat Mass Index (FMI) was calculated as the Fat Mass (kg) divided by the square of height (m^2^).(15)

### Resting pulse rate and arterial blood pressure recording

Resting pulse rate (PR) was used as an index of cardiac autonomic balance with a higher PR indicative of cardiac sympathetic overactivity.

Participant condition: The participants were asked to avoid the intake of any stimulant (drugs, coffee etc) for a period of at least 30 min before the measurement. The participants were also asked to empty the bladder before the measurement and relax quietly in sitting position for a period of at least 5 min. The PR and blood pressure were measured in the left upper extremity in sitting position with arm and back support, uncrossed legs and feet on the floor.(16,17)

The Pulse rate (PR), Systolic blood pressure (SBP) and Diastolic blood pressure (DBP) were measured at the brachial artery from the left arm using the Omron T8 (HEM757A4-C1) Automatic Blood Pressure instrument (Accuracy, BP: ±4 mm Hg, Pulse: ±5), validated by Association for the Advancement of Medical Instrumentation, AAMI and British Hypertension Society, BHS).(18) Pulse rate and blood pressure were recorded at intervals of 1 minute till the difference between two consecutive BP readings was less than 5 mm Hg. The average of the two consecutive readings was used for statistical analysis.(16,17)

Pulse pressure (PP) and mean arterial pressure (MAP) were calculated from the average values of SBP and DBP using the formula shown below:

PP = SBP - DBP

MAP = DBP + 1/3(PP)

### Cardiovascular reactivity to acute sympathetic stress

The cardiovascular response to acute sympathetic stress was assessed by Isometric Hand Grip test (Sustained Hand Grip test). Isometric handgrip test evaluates the cardiovascular adrenergic function and is recommended to be used as an investigational autonomic function test by the American Academy of Neurology.(19) The participants were asked to do maximum voluntary contraction using the handgrip dynamometer with the dominant hand. Three attempts were made at intervals of 1 minute and the highest reading amongst the attempts was considered as the maximum voluntary contraction (MVC) for the participant. Pre-exercise PR and blood pressure were measured prior to exercise at the brachial artery from the arm not involved in contraction (non-dominant arm) using the Omron T8 Automatic Blood Pressure instrument. The participants were then asked to perform isometric handgrip exercise at an intensity of 30% MVC for 1 minute in the sitting position posture. Pulse rate and Blood Pressure were measured at 1 minute of exercise at the brachial artery from the non-dominant arm.(20) The Percentage Rise in DBP (%RDBP) was calculated from the pre-exercise and 1-minute exercise values of DBP as a function of cardiovascular reactivity to acute sympathetic stress.

Statistical analysis: The subjects were categorized into two groups based on the sleep duration at night: 1) Adequate Sleep Duration at Night, ASDN (≥7 hrs) and 2) Inadequate Sleep Duration at Night, ISDN (<7 hrs).(8,9) The dependent variables (Physical Activity Status, Body Composition, Resting PR, Blood Pressure Profile and Cardiovascular Reactivity to Stress) of the two groups were then compared using Unpaired Student's *t*-test at 95% confidence limit and 5% confidence interval.

## Results

Tables 1 and 2 indicate that in both boys and girls, respectively, PR, SBP, DBP, PP and MAP did not differ significantly between adolescents having adequate sleep duration at night and those who slept inadequately at night. Physical activity status also did not seem to differ significantly between the two groups. However, adolescents with inadequate sleep did have a significantly higher level of adiposity and cardiovascular reactivity in comparison to those adolescents who slept adequately as indicated by the significant differences in BMI, BF%, FM, FMI, WC and %RDBP between the two groups.

**Table 1:** Baseline characteristics of Gujarati adolescent boys with adequate sleep duration and inadequate sleep duration at night

Study variable	ASDN(*N* = 210)	ISDN(*N* = 87)
SDN	7.9 ± 1.1	5.7 ± 0.48**
PA-R	3 ± 1.2	3.2 ± 1.5
Weight (kg)	49 ± 10.5	56 ± 12.3**
Height (m)	1.64 ± 0.08	1.67 ± 0.07*
Body mass index, BMI (kg/m^2^)	18.2 ± 2.8	19.8 ± 3.8**
Body fat %	16 ± 5.2	17.6 ± 5.8*
Fat mass (kg)	8.3 ± 4.1	10.3 ± 5.4**
Fat mass index, FMI (kg/m^2^)	3 ± 1.4	3.6 ± 1.9**
Waist circumference, WC (cm)	65.3 ± 6.8	68.8 ± 9.4**
Pulse rate (pulse/min)	80 ± 12	82 ± 10.9
Systolic blood pressure (mmHg)	116 ± 10	116 ± 10.5
Diastolic blood pressure (mmHg)	73 ± 7.7	74 ± 7.4
Pulse pressure (mmHg)	42 ± 8	42 ± 7.4
Mean arterial pressure (mmHg)	88 ± 7.7	88 ± 7.8
%RDBP(mmHg)	19.5 ± 12(182)	23.13 ± 14.7 (72)*

Values are Mean±SD

* *P*<0.05

** *P*<0.01. ASDN – Adequate sleep duration at night (≥7 hrs), ISDN – Inadequate sleep duration at night (<7 hrs), PA-R- Physical activity rating, %RDBP – Percentage rise in DBP due to sympathetic stress. Figures in bracket indicate number of subjects

**Table 2:** Baseline characteristics of Gujarati adolescent girls with adequate sleep duration at night and inadequate sleep duration at night

Study variable	ASDN (N=139)	ISDN (N=53)
SDN	7.6 ± 0.8	5.8 ± 0.3*
PA-R	1.96 ± 0.98	1.98 ± 1.23
Height (m)	1.54 ± 0.06	1.55 ± 0.06
Body mass index, BMI (kg/m^2^)	19 ± 3.5	20.4 ± 3.5*
Body fat %	23.3 ± 7	26.2 ± 5.7*
Fat mass (kg)	11.2 ± 5.6	13.4 ± 5.4*
Fat mass index, FMI (kg/m^2^)	4.6 ± 2.2	5.5 ± 2.1*
Waist circumference, WC (cm)	63.5 ± 7	64.9 ± 7.2
Pulse rate (pulse/min)	88 ± 12.7	87 ± 16.8
Systolic blood pressure (mmHg)	112 ± 8.9	112 ± 9.7
Diastolic blood pressure (mmHg)	76 ± 7.9	77 ± 8
Pulse pressure (mmHg)	36 ± 7.4	35 ± 5.7
Mean arterial pressure (mmHg)	62 ± 6.8	61 ± 6.2
%RDBP (mmHg)	14 ± 9(125)	21 ± 11.9 (48)*

Values are Mean±SD

* *P*<0.05. ASDN – Adequate sleep duration at night (≥7 hrs), ISDN – Inadequate sleep duration at night (<7 hrs), PAR – Physical activity rating, %RDBP – Percentage rise in DBP due to sympathetic stress. Figures in bracket indicate number of subjects

## Discussion

Our study failed to show any significant effect of sleep duration on the blood pressure profile of the Gujarati Indian adolescents. This is, however, contradictory to the findings of research conducted on children and adolescents in other parts of the world. Sampei, Murata, Dakeishi and Wood reported the relationship between Total Sleep Duration (TSD) and blood pressure amongst Japanese children of age group 5–6 years. They found that TSD had a significant correlation (*r* = 0.265) with SBP but not with DBP.(21)

Javaheri, Storfer-Isser, Rosen and Redline studied the relationship of inefficient sleep with prehypertension amongst healthy U.S. adolescents.(5) Analysis of their study revealed that after adjusting for gender, BMI and socioeconomic status, the odds of prehypertension increased 3.5 fold for low sleep efficiency and 2.5 fold for short sleep. The study showed that adolescents with low sleep efficiency had on average a 4.0 ± 1.2 mm Hg higher SBP than those who had a better sleep efficiency.

Mei-Yen Chen, Edward K Wang and Yi-Jong Jeng reported that sleep deprivation impairs physical functioning and emotional well-being with reduction in motivation and early fatigue amongst adolescents.(22)

Thus the differences between our findings and earlier research work need to be understood. We had believed and hypothesized that sleep duration would probably affect the physical activity status, adiposity, autonomic balance and/or cardiovascular reactivity to stress and thereby affect the blood pressure as indicated by previous studies conducted on adolescents and adults.(22–24)

An important finding in our study, which goes in line with our view that sleep deprivation may not be associated with blood pressure in this population, is that adolescents with inadequate sleep are found to be involved in equal amount of physical activity as compared to those adolescents who have adequate sleep. Therefore, due to involvement in physical activity, the sleep-deprived adolescents may be maintaining their blood pressure at a level, which is similar to those adolescents who are sleeping adequately.

Another finding that supports our view is that the sympathetic activity does not seem to be affected by sleep duration as indicated by the indifference observed between the PR of adolescents with adequate sleep and those with inadequate sleep. Henceforth, sleep deprivation may not affect the blood pressure. This finding is again contradictory to the earlier research work, which indicates that sleep deprivation is associated with sympathetic overactivity as indicated by increase excretion of urinary catecholamines in subjects who were deprived of sleep and had higher blood pressure levels in comparison to those subjects who got adequate sleep.(24) Although contradictory to earlier findings, both these findings support our view that blood pressure of the Gujarati Indian adolescents is not affected by sleep duration in this age group.

However, there are two other important observations in our study which do not favor our view. First, sleep duration shows a significant effect on the adiposity of the adolescents, such that adolescents with inadequate sleep have a higher adiposity as compared to those who slept adequately. Thus, this finding does not support the current result because earlier research indicates that increase adiposity associated with sleep deprivation leads to hypertension.(24) Sleep deprivation causes obesity by affecting the balance of Leptin and Ghrelin concentration, appetite and insulin sensitivity. But, studies also reveal direct effects of sleep deprivation on the blood pressure level independent of body weight.(6) Therefore, it is thus possible that the increase in adiposity observed in adolescents with inadequate sleep may not be large enough to cause a rise in blood pressure amongst these adolescents.

The second important observation that does not favor the current result is that inadequate sleep duration increases the cardiovascular reactivity to stress. Increase cardiovascular reactivity to stress has been linked to hypertension in previous studies.(25) But, it is again possible that the difference in cardiovascular reactivity due to difference in sleep duration though significant may not be large enough to cause differences in blood pressure profile amongst the Gujarati Indian adolescents.

However, apart from all these complex observations of our study, we believe that the observed insignificant effect of sleep duration on the resting cardiac autonomic balance as indicated by the resting PR in this population is indeed probably the major reason due to which the blood pressure profile of the adolescents does not get affected despite having inadequate sleep duration.

## Conclusion

The current study indicates that inadequate sleep duration at night (<7 hrs) does not affect the blood pressure profile of the Gujarati Indian adolescents of age group 16–19 years despite causing a significant increase in adiposity and cardiovascular reactivity to stress. This is probably because inadequate sleep does not affect the cardiovascular autonomic balance at rest. However, longitudinal studies would be required to observe the impact of inadequate sleep durations during childhood and adolescence on the blood pressure profile during the later life.

### Limitations and future perspectives

A major limitation of the study is that, it is a cross-sectional study and that the sleep duration and physical activity status have been assessed subjectively. Therefore, although this study gives significant information about the subject under consideration, longitudinal and/or experimental studies involving more objective measures and biochemical parameters are required to assess the actual causal relationship between sleep duration and blood pressure.
